# Assessment of the mental workload of trainee pilots of remotely operated aircraft using functional near-infrared spectroscopy

**DOI:** 10.1186/s12883-022-02683-5

**Published:** 2022-04-30

**Authors:** Liya Tang, Juanning Si, Lei Sun, Gengsheng Mao, Shengyuan Yu

**Affiliations:** 1 Department of Neurology, the First Medical Centre of Chinese PLA General Hospital, Haidian District, No.28 Fuxing Road, Beijing, 100089 China; 2 School of Instrumentation Science and Opto-Electronics Engineering, Beijing Information Science and Technology University, Beijing, China; 3 Department of Biomedical Engineering, The HongKong Poly Hung Home, HongKong Special Administrative Region, Hung Hom, Hong Kong; 4 Department of Neurovascular Surgery, the Third Medical Centre of Chinese, PLA General Hospital, Beijing, China

**Keywords:** Brain, Deoxyhemoglobin, Flight performance, Functional near-infrared spectroscopy, fNIRS, Mental workload, Remotely operated vehicle operators, Oxyhemoglobin

## Abstract

**Background:**

Operating an aircraft is associated with a large mental workload; however, knowledge of the mental workload of ROV operators is limited. The purpose of this study was to establish a digital system for assessing the mental workload of remotely operated vehicle (ROV) operators using hemodynamic parameters, and compare results of different groups with different experience levels.

**Method:**

Forty-one trainee pilots performed flight tasks once daily for 5 consecutive days in a flight simulation. Forty-five pilots experienced pilots and 68 experienced drivers were also included. Hemodynamic responses were measured by functional near-infrared spectroscopy (fNIRS).

**Results:**

The median duration of peak oxyhemoglobin was 147.13 s (interquartile range [IQR] 21.97, 401.70 s) in the left brain and 180.74 s (IQR 34.37, 432.01 s) in the right brain in the experienced pilot group, and 184.42 s (IQR 3.41, 451.81 s) on day 5 in the left brain and 160.30 s (IQR 2.62, 528.20 s) in the right brain in the trainee group.

**Conclusion:**

Navigation training reduces peak oxyhemoglobin duration, and may potentially be used as a surrogate marker for mental workload of ROV operators. Peak oxyhemoglobin concentration during s task may allow development of a simplified scheme for optimizing flight performance based on the mental workload of a pilot.

**Supplementary Information:**

The online version contains supplementary material available at 10.1186/s12883-022-02683-5.

## Introduction

Operating an aircraft imposes a daunting physical and mental workload on pilots. The recent introduction of highly automated vehicles and remotely operated vehicles (ROV), has reduced the physical workload of operators but has dramatically increased the mental workload, including that of drone operators [1, 2]. This increase of mental demands to pilot ROVs may compromise operator task performance.

Kramer and Parasuraman define mental workload or cognitive load as a set of mental and composite brain states that modulate human performance in different perceptual, cognitive, and/or sensorimotor skills [3]. Mental workload is a key determinant of, and positively correlated with, operator task performance [4]. Morris and Leung reported that increased mental workload significantly hindered task execution of trainee pilots [5]. Svensson et al. showed that pilot mental workload affected both objective and subjective aspects of performance [6]. As such, it is critical that the mental workload of aircraft operators be optimized so that operator performance is maintained and human errors during flight operation are minimized [7].

Mental workload has been actively investigated over the prior decade using the electroencephalogram (EEG), event-related potential (ERP), heart rate and respiration variability, and functional near-infrared spectroscopy (fNIRS) [8–10]. However, most prior studies were conducted in a virtual or real working environment of experienced automobile drivers and airplane pilots; the level of mental workload required to operate ROVs and correlates have not been fully investigated.

fNIRS measures changes in cerebral blood flow and oxyhemoglobin and deoxyhemoglobin concentrations using near-infrared detectors placed on the scalp, and has shown that oxyhemoglobin concentrations increase with increasing memory load [11]. Durantin et al. studied the mental workload of 12 volunteers who performed a computer-based piloting task, and showed that fNIRS and heart rate variability could predict the level of mental workload [8].

In the current study, we establish a digital system for the assessment of the mental workload of trainee pilots of ROVs based on fNIRS. In addition, the mental workload of experienced pilots and experienced drivers was assessed, and the results of the 3 groups were compared.

## Methods

### Subjects

The study was approved by the ethics committee at the authors’ institution. All study participants provided written informed consent for participation in the study. Forty-five experienced pilots with a median experience level of 15 years (interquartile range [IQR]: 3, 26 years) and 68 experienced drivers (mean age 23.4 ± 3.3 years) were included. In addition, 41 trainee pilots (mean age 21.1 ± 2.1 years) with no previous experience in operating aircrafts were also included.

All subjects were evaluated using the Symptom Checklist-90 (SCL90) questionnaire, the Hamilton Anxiety Scale (HAM-A), and Hamilton Depression Scale (HAM-D) to exclude subjects with psychological abnormalities. The trainee pilots and experienced drivers were trained to remotely operate an aircraft in a straight line between cities. Subjects who achieved a ratio greater than 0.8 of the simulated straight-line distance to the actual flight distance using the Smith Visual Space Test system were eligible to participate in the study and undergo formal testing.

### Drone operating procedure

An operating module with a 1:100 operator space (100 × 100 × 100 m^3^) was designed, and 4 fixed sites were placed at different positions in the operating space. The 4 sites represented 4 cities (Beijing, Xian, Jinan, and Shenyang, China) and were connected by straight lines. The operator joystick was adjusted according to hand dominance. All subjects wore an 8-lead, portable fNIRS brain monitor [12].

In the first phase, when a plus ( +) sign appeared in the center of the screen and became stable, the subject began to remotely operate the aircraft. For the first 60 s, the subject remotely flew the aircraft in an empty space at will in order to become familiarized with the operating environment. The subjects were then asked to remember the locations of the 4 cities. In the seconds phase, flight tasks (flying to the 4 cities) appeared randomly on the screen, and the subjects were required to complete the tasks using the joystick to operate the aircraft. There was no time requirement for completing the individual tasks, and there were no changes once a task was begun.

**Fig. 1:**
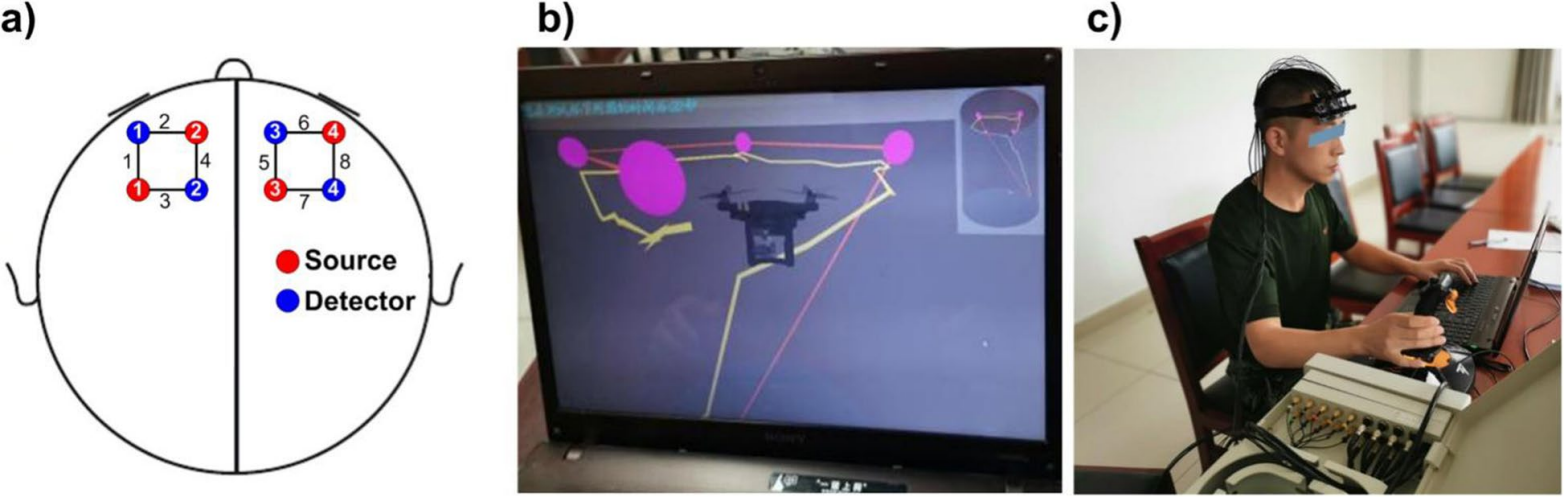
**A** Flight space of the UAV stimulated by the space navigation system (from top to bottom); **B** The computer software automatically calculates the best flight routes. **C** Image of a subject wearing the fNIRS detection instrument and operating the joystick during a simulation

In the third phase, the system was reset and the subjects were required to complete the flight tasks in a straight line along the memorized routes (flying from one destination to the next). A 5-s minimum stopover at each destination was required. If the operator correctly completed the scheduled flight routes, after departure from the final destination the flight time was recorded as the flight performance score, and actual flight routes and computer simulated optimal routes were displayed on the screen (Fig. 1A, B).

If there was an error in the scheduled flight routes, the trainee repeated the flight task until it was completed error-free. The subjects performed the flight tasks once daily for 5 consecutive days, and were evaluated using the NASA Task Load Index [13]. The Index consists of 6 items that are subjectively evaluated on a scale. After each flight, the operators were scored using the Index. The total task duration was defined as the time the flight operator entered the operating module to the completion of the flight task, and included the time required for mission assignment and flight planning.

### Flight preparation and flight performance

Flight preparation time: The subject controls the drone and flies it freely in order to understand the spatial conditions of the flight area. After 1 min, the computer software stops the free flight and gives a random sequence of flight missions. The operator designs the actual flight route, and then presses "OK" to start the mission and start recording the time.

Flight performance: The time from the subject pressing "OK" to completing the flight mission correctly was recorded in seconds, and was defined as flight performance.

### fNIRS recording and data analysis

Hemodynamic responses of the subjects were measured using a custom-designed, portable fNIRS system [12]. Two pads with optodes were placed on the scalp above the prefrontal area (Fig. 1C). Each pad contained 2 sources and 2 detectors, yielding 4 optical channels. The distance between the source and detector pairs was 30 mm, and each probe pad covered an area of approximately 30 × 30 mm^2^. The sampling rate of the fNIRS system was 100 Hz, and the hemodynamic responses were quantitatively analyzed. fNIRS data were analyzed using the MATLAB 2019a platform (Mathworks Inc., Natick, MA, USA).

First, the relative concentration changes of oxyhemoglobin, deoxyhemoglobin, and total hemoglobin were calculated from the raw optical data based on the modified Beer-Lambert law (MBLL) [14]. The differential path length factors (DPF) for 690 nm and 830 nm were 6.51 and 5.86, respectively [15]. Then, the hemodynamic data were low-pass filtered with a third-order Butterworth filter. The cutoff frequency of the filter was set to 0.3 Hz to reject task-unrelated noise due to heartbeat, breathing, and high frequency instruments. Thereafter, the filtered hemodynamic data were extracted beginning 5 s before activation onset and ending after the task was completed. Data with large artifacts were discarded.

After baseline correction (the 5-s period before activation onset was regarded as the baseline), the hemodynamic response functions were calculated for further analysis. First, the mean hemodynamic responses of the left hemisphere (channel 1, 2, 3, and 4) and the right hemisphere (channel 5, 6, 7, and 8) were calculated. Then, the peak and mean values of the mean hemodynamic responses were calculated during the preparation stage and the flight stage. Therefore, only 1 peak was calculated for each hemisphere.

A typical brain activation was characterized by a significant increase in oxyhemoglobin concentration or a significant decrease in deoxyhemoglobin concentration as compared to the baseline after activation [16]. Preliminary study showed that fNIRS results indicated that both oxyhemoglobin and deoxyhemoglobin levels in the left or right brain increased from the preparation stage to the actual flight stage.

Therefore, values were measured at the preparation stage and during the whole flight. In addition, the peak and mean values during the task period of each trainee pilot were extracted from the data channels for comparisons between different conditions.

**Fig. 2:**
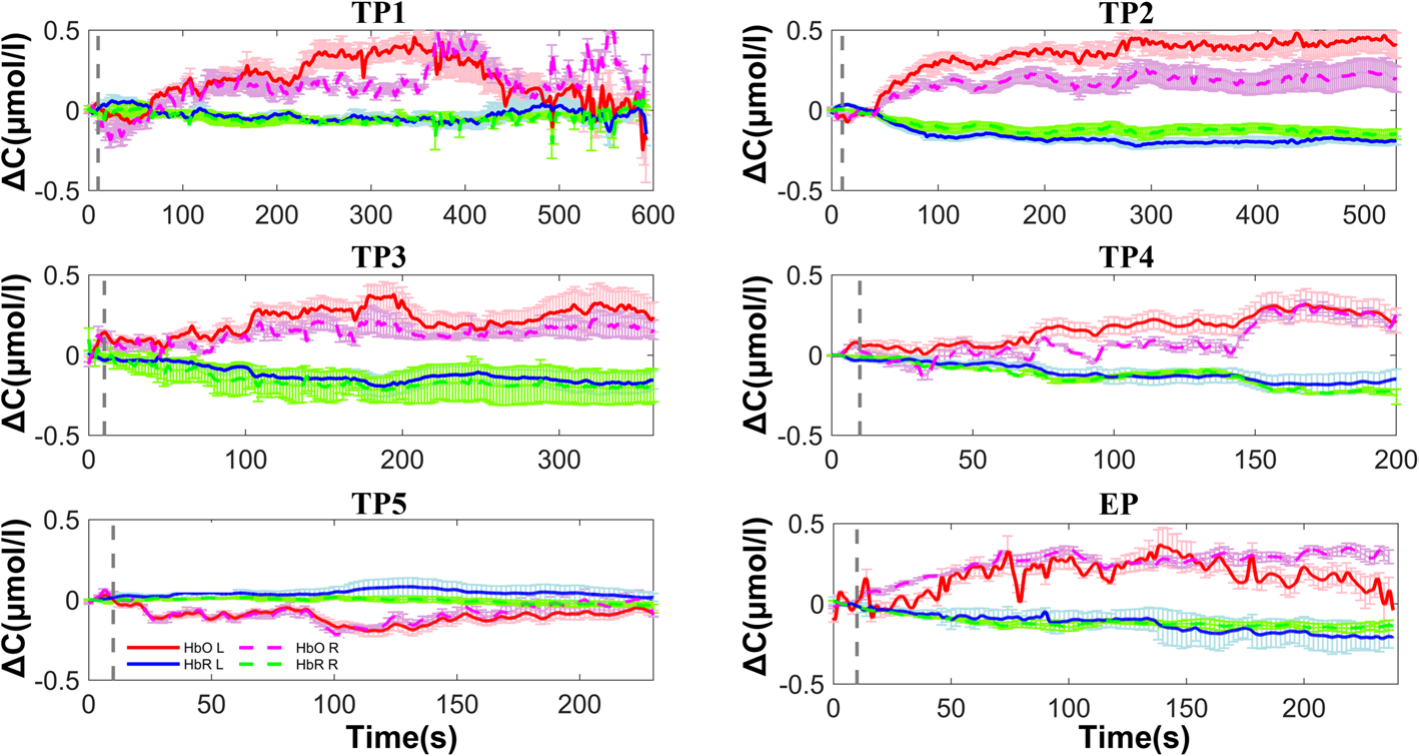
Oxyhemoglobin and deoxyhemoglobin levels in the left and right brain from day 1 to day 5 in the trainee pilot group vs. the experienced pilot group. TP = trainee pilot; EP = experienced pilot; HbO = oxyhemoglobin; HbR = deoxyhemoglobin; L = left brain; R = right brain. Number associated with TP indicates day, i.e., TP1 = trainee pilot day 1, etc

**Fig. 3:**
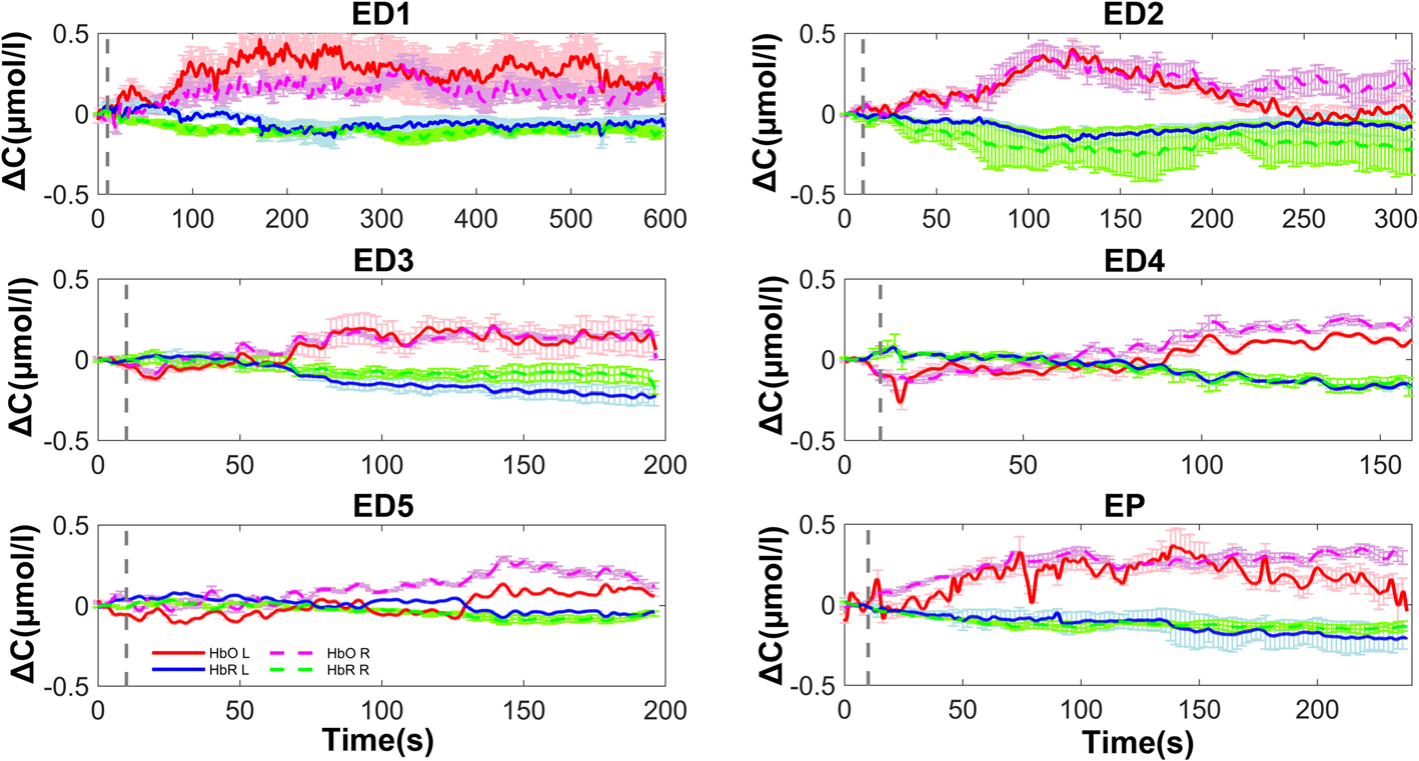
Oxyhemoglobin and deoxyhemoglobin levels in the left and right brain from day 1 to day 5 in the experienced driver group vs. the experienced pilot group. ED = experienced driver; EP = experienced pilot; HbO = oxyhemoglobin; HbR = deoxyhemoglobin; L = left brain; R = right brain. Number associated with ED indicates day, i.e., ED1 = experienced driver day 1, etc

Results that illustrate the recordings are shown in Fig. 2 and Fig. 3.

## Statistical analysis

Data were expressed as mean ± standard deviation, or median [IQR]. Comparisons between the experienced drivers group and the experienced pilots group, and between the trainee pilots group and the experienced drivers group, were performed with the t-test. Comparisons of measurements between different times within groups were examined by repeated measures ANOVA, with post hoc tests. Pearson correlation analysis was conducted to characterize the relations between measured variables. A value of *p* < 0.05 was considered to indicate statistical significance. Statistical analyses were performed using SPSS 22 statistical software (SPSS Inc., Chicago, IL, USA).

## Results

### Total task duration and flight preparation

The mean total task duration was 342.41 ± 152.66 s in the experienced pilot group. The mean total task duration was 463.59 ± 195.73 s on day 1 in the trainee pilot group, and declined to 288.94 ± 150.46 s on day 5, which was significantly shorter than the duration on day 1 (*p* < 0.001). In the experienced driver group, the mean total task duration was 574.06 ± 211.33 s on day 1, and decreased to 225.90 ± 95.18 s on day 5 (*p* < 0.001).

The total time of trainee pilots and experienced drivers for task preparation was significantly longer than that of experienced pilots on the first day (t test; *p* = 0.016 for experienced pilots vs. trainee pilots, and *p* = 0.000 for experienced pilots vs. drivers, respectively). After repeated simulations, the time for task preparation was significantly decreased for trainee pilots and experienced drivers, and was significantly shorter than that of experienced pilots on day 4 (t test; *p* = 0.014 for experienced pilots vs. trainee pilots *p* = 0.029 for experienced pilots vs. drivers, respectively).

### Flight performance

The mean flight performance score (time to complete the task in seconds; a lower score indicates better performance) of the experienced pilot group was 179.37 ± 82.47 s, and of the trainee pilot group was 252.67 ± 152.29 s on day 1; significantly higher than that of experienced pilots (t test; *p* = 0.001). The score in the trainee pilot group steadily declined thereafter, and was the lowest on day 4 (136.19 ± 102.80 s), and the value was significantly lower than that on day 1 (*p* = 0.000). The mean flight performance score of the experienced driver group was 408.07 ± 208.82 s on day 1, and was significantly higher than that of experienced pilots (t test; *p* = 0.006). The mean experienced driver then score gradually declined, and was the lowest on day 5 (136.70 ± 78.90 s), which was significantly lower than the score on day 1 (*p* < 0.001).

**Fig. 4:**
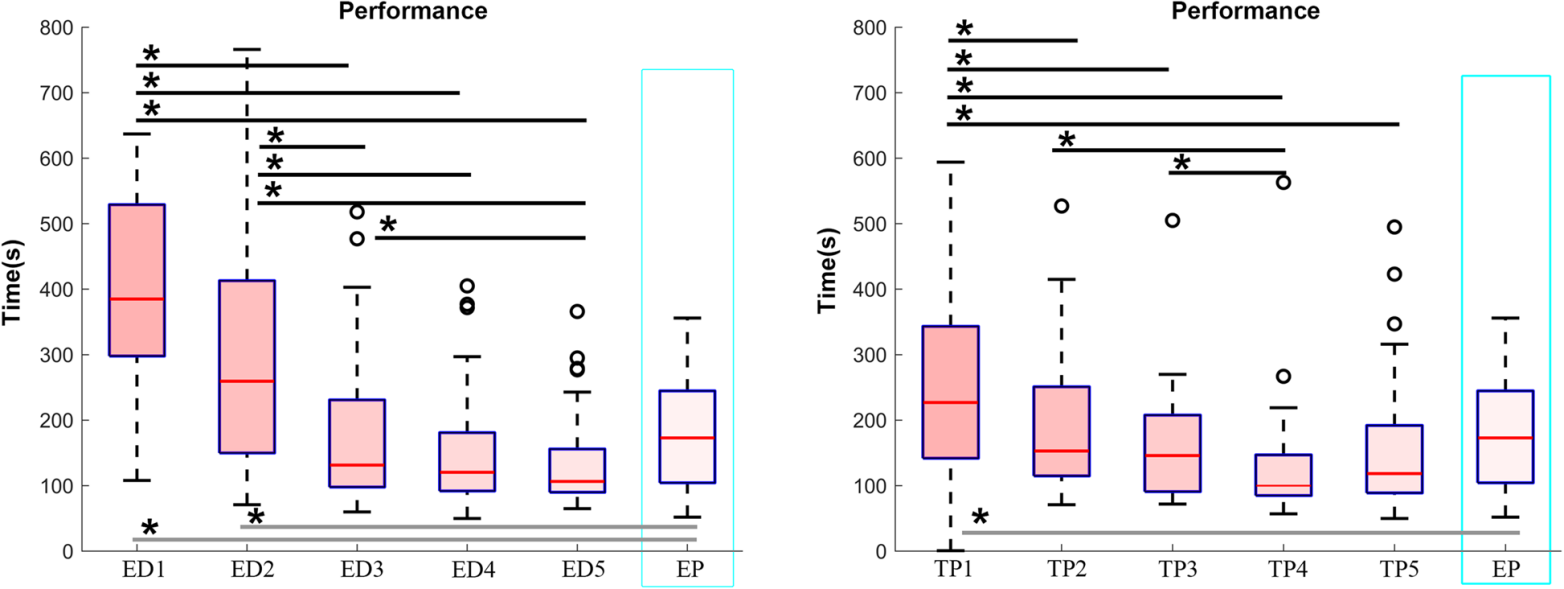
Flight performance (total flight duration in seconds) of the trainee pilot group (left) and experienced driver group (right) from day 1 to day 5. ED = experienced driver; TP = trainee pilot; EP = experienced pilot. Number associated with ED or TP indicates day, e.g., ED1 = experienced driver day 1, etc. *, *p* < 0.05 compared with EP

Compared with experienced pilots, the flight time of trainee pilots and experienced drivers was significantly higher than that of pilots on the first day (t test; *p* = 0.006 vs. trainee pilots; *p* = 0.000 vs. experienced drivers). After repeated simulations, the flight time of experienced drivers was markedly shorter than that of experienced pilots on day 5 (*p* = 0.05) (Fig. 4). In summary, on the first day the time to complete the task of trainee pilots and experienced drivers was higher than that of experienced pilots. On the fourth and fifth days, the time to complete the task of the trainee pilots and experienced drivers was less than that of experienced pilots, indicating that performance was improved with practice.

### Total hemoglobin

#### Experienced pilots

In the experienced pilot group, the total hemoglobin concentration was 0.01 ± 0.16 µmol/L in the left brain and 0.04 ± 0.07 µmol/L in the right brain. The peak total hemoglobin concentration was 0.30 ± 0.36 µmol/L in the left brain and 0.18 ± 0.11 µmol/L in the right brain. The duration of peak oxyhemoglobin concentration was 162.77 ± 95.47 s in the left brain and 194.30 ± 109.52 s in the right brain.

#### Trainee pilots

**Fig. 5:**
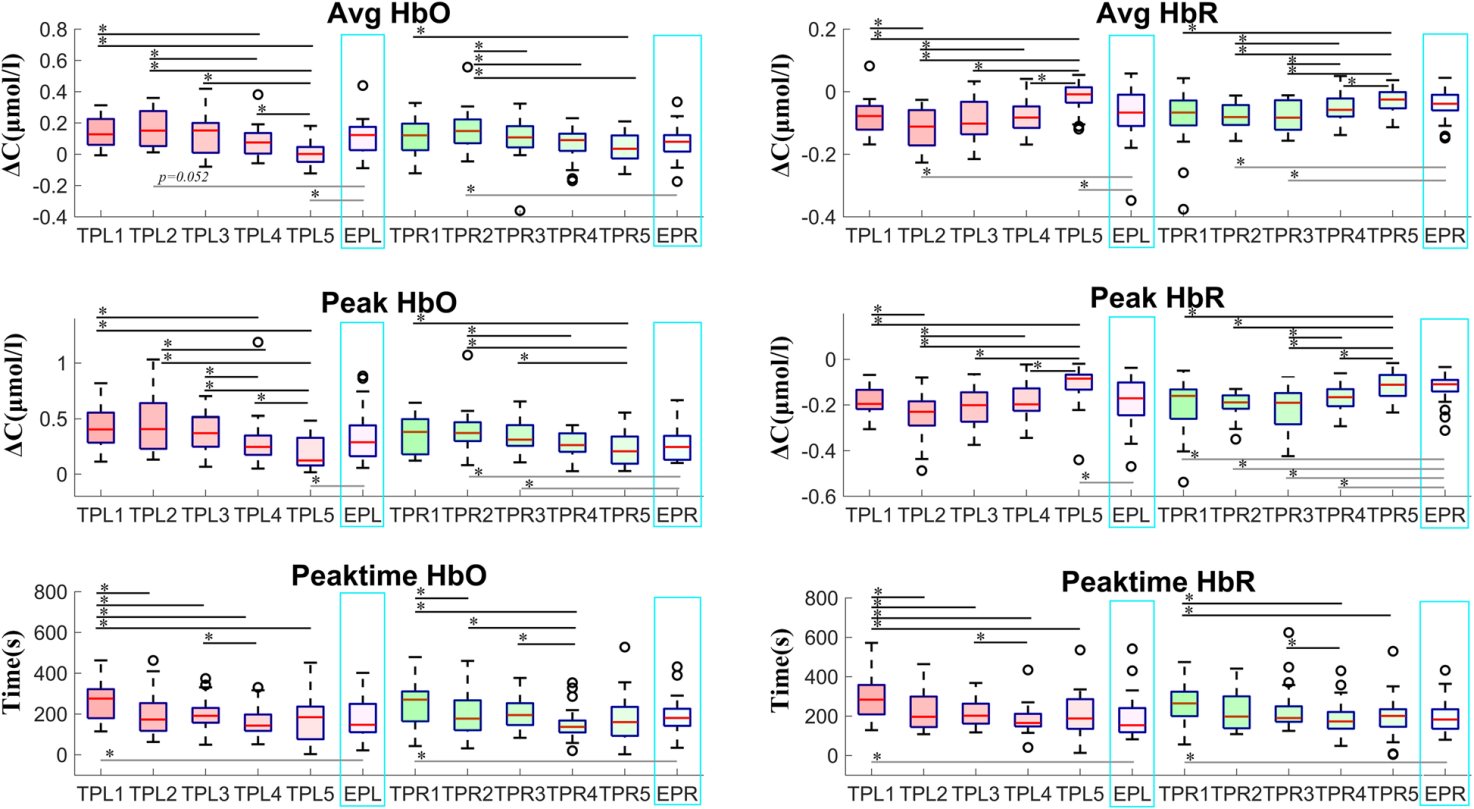
Mean and peak concentration of oxyhemoglobin and deoxyhemoglobin, and duration of peak concentrations in the left and right brains of the trainee pilot group and the experienced pilot group from day 1 to day 5. TP = trainee pilot; TPL = left brain of trainee pilot; TPR = right brain of trainee pilot; EPR = right brain of experienced pilot; EPL = left brain of experienced pilot. Numbers following TPL, TPR, EPR, and EPL indicate day of testing. *, *p* < 0.05

In the trainee pilot group, the total hemoglobin concentration in the left brain was 0.06 ± 0.08 µmol/L on day 1, and declined to -0.01 ± 0.05 µmol/L on day 5 (*p* = 0.001). In the right brain, it was 0.04 ± 0.13 µmol/L on day 1 and declined to 0.01 ± 0.07 µmol/L on day 5. The peak total hemoglobin concentration in the left brain was 0.28 ± 0.19 µmol/L on day 1, and decreased to 0.13 ± 0.13 µmol/L on day 5 (*p* < 0.001). In the right brain, it was 0.39 ± 0.71 µmol/L on day 1 and declined to 0.16 ± 0.11 µmol/L on day 5. The duration of peak total hemoglobin concentration in the left brain decreased from 230.39 ± 134.46 s on day 1 to 138.50 ± 99.65 s on day 5 (*p* = 0.015), and from 238.97 ± 118.18 s on day 1 to 141.22 ± 98.36 s on day 5 in the right brain (*p* = 0.009). After the first practice, the duration of peak total hemoglobin concentration in trainee pilots was significantly longer than that of experienced pilots (t test; left, *p* = 0.041). After repeated testing, the peak time decreased and at the 4^th^ practice the peak time of total hemoglobin concentration in the right brain of trainee pilots was significantly shorter than that of experienced pilots (*p* = 0.026) (Fig. 2, Fig. 5).

#### Experienced drivers

In the experienced driver group, the total hemoglobin concentration in the left brain was 0.04 ± 0.13 µmol/L on day 1, and decreased to -0.04 ± 0.12 µmol/L on day 5. In the right brain it was 0.04 ± 0.15 µmol/L on day 1 and decreased to -0.03 ± 0.09 µmol/L on day 5 (*p* = 0.019). The peak total hemoglobin concentration in the left brain was 0.31 ± 0.14 µmol/L on day 1, and decreased to 0.15 ± 0.15 µmol/L on day 5 (*p* < 0.001). In the right brain it was 0.36 ± 0.22 µmol/L on day 1 and declined to 0.26 ± 0.18 µmol/L on day 5. The duration of peak total hemoglobin concentration in the left brain was 270.02 ± 193.95 s on day 1 and decreased to 95.17 ± 73.06 s on day 5 (*p* < 0.001). In the right brain it was 298.83 ± 246.15 s on day 1 and 126.52 ± 72.51 s on day 5 (*p* = 0.001).

**Fig. 6:**
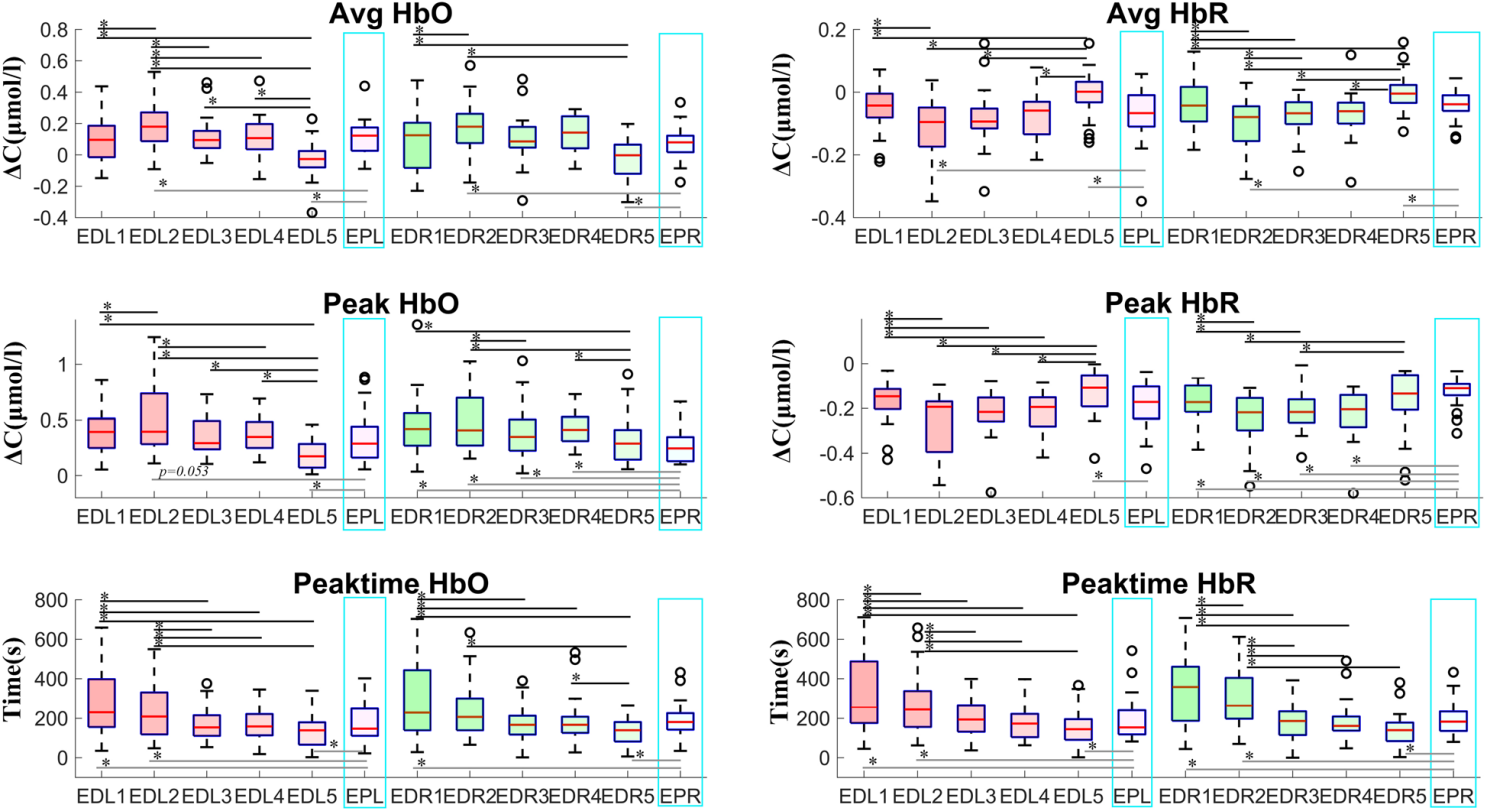
Mean and peak concentration of oxyhemoglobin and deoxyhemoglobin, and duration of peak concentrations in the left and right brains of the experienced driver group and the experienced pilot group from day 1 to day 5. ED = experienced driver; EDL = left brain of experienced driver; EDR = right brain of experienced driver; EPR = right brain of experienced pilot; EPL = left brain of experienced pilot. Numbers following EDL, EDR, EPR, and EPL indicate day of testing. *, *p* < 0.05

The median peak total hemoglobin concentration and the median duration of peak total hemoglobin in the left brain of experienced drivers on day 1 was markedly longer than that of experienced pilots (t test; *p* = 0.014, *p* = 0.018, respectively). Notably, the total peak oxyhemoglobin concentration in the left brain of experienced drivers during the fourth and the fifth flight mission was significantly lower than that of experienced pilots (t test; *p* = 0.027, *p* = 0.014, respectively). In the right brain, the median total hemoglobin concentration, the median peak total hemoglobin concentration, and the median duration of peak total hemoglobin concentration on day 1 was markedly longer than that of experienced pilots (t test; *p* = 0.000, *p* = 0.009, *p* = 0.005, respectively). The median peak time of total hemoglobin was significantly different from that of experienced pilots on day 1 (t test; left, *p* = 0.010; right, *p* = 0.041). After repeated simulations, on day 5 the median peak time of total hemoglobin was markedly shorter than that of the pilots (left, *p* = 0.005; right, *p* = 0.009). In addition, after repeated simulations, the mean and peak total hemoglobin concentrations in the right brain were significantly lower than those of experienced pilots (mean, *p* = 0.005; peak, *p* = 0.022) (Fig. 3, Fig. 6).

### Oxyhemoglobin

#### Experienced pilots

In the experienced pilot group, the oxyhemoglobin concentration on day 1 in the left brain was 0.08 ± 0.18 µmol/L, and was 0.08 ± 0.10 µmol/L in the right brain. The peak oxyhemoglobin concentration in the left brain was 0.37 ± 0.31 µmol/L, and in the right brain was 0.25 ± 0.14 µmol/L. The duration of peak oxyhemoglobin concentration was 183.47 ± 90.48 s in the left brain and 191.77 ± 85.74 s in the right brain.

#### Trainee pilots

In the trainee pilot group, the oxyhemoglobin concentration in the left brain on day 1 was 0.14 ± 0.09 µmol/L, and declined to 0.01 ± 0.08 µmol/L on day 5 (*p* < 0.001). In the right brain it was 0.11 ± 0.12 µmol/L on day 1, and declined to 0.04 ± 0.09 µmol/L on day 5 (*p* = 0.010). The peak oxyhemoglobin concentration in the left brain was 0.40 ± 0.19 µmol/L on day 1, and decreased to 0.19 ± 0.15 µmol/L on day 5 (*p* < 0.001). In the right brain it was 0.44 ± 0.50 µmol/L on day 1 and declined to 0.23 ± 0.16 µmol/L on day 5 (*p* = 0.026). The duration of peak oxyhemoglobin concentration in the left brain was 283.86 ± 125.74 s on day 1 and 169.64 ± 112.10 s on day 5 (*p* = 0.004). In the right brain it was 249.87 ± 108.60 s on day 1 and 181.25 ± 120.12 s on day 5 in the right brain (*p* = 0.026). As compared to experienced pilots, the mean and peak oxyhemoglobin concentrations in the left brain on day 5 were markedly lower than those of the pilots (mean, *p* = 0.046; peak, *p* = 0.010) (Fig. 2, Fig. 5).

#### Experienced drivers

In the experienced driver group, the oxyhemoglobin concentration in the left brain was 0.09 ± 0.18 µmol/L on day 1, and decreased to -0.03 ± 0.12 µmol/L on day 5 (*p* = 0.003). In the right brain it was 0.08 ± 0.19 µmol/L on day 1 and -0.02 ± 0.12 µmol/L on day 5 (*p* = 0.003). The peak oxyhemoglobin concentration in the left brain was 0.40 ± 0.21 µmol/L on day 1 and 0.19 ± 0.13 µmol/L on day 5 (*p* < 0.001). In the right brain it was 0.44 ± 0.26 µmol/L on day 1 and 0.31 ± 0.21 µmol/L on day 5 (*p* = 0.024). The duration of peak oxyhemoglobin concentration in the left brain was 284.31 ± 186.05 s on day 1 and 134.44 ± 83.53 s on day 5 (*p* < 0.001). In the right brain it was 308.59 ± 244.63 s on day 1 and 130.16 ± 71.32 s on day 5 (*p* = 0.001).

The median duration of peak oxyhemoglobin concentration in the left and right brain of experienced drivers on day 1 was significantly longer than that of experienced pilots (t test; *p* = 0.011, *p* = 0.019, respectively). The mean oxyhemoglobin concentration, median peak concentration, and peak duration of oxyhemoglobin concentration in the left brain on day 5 was significantly lower than that of experienced pilots (t test; *p* = 0.007, *p* = 0.007, *p* = 0.039, respectively). In the right brain, the peak oxyhemoglobin concentration and duration on day 1 was significantly greater than that of experienced pilots (t test; *p* = 0.001, *p* = 0.019, respectively). After repeated testing, the peak concentration of oxyhemoglobin and duration of peak concentration in the right brain was significantly lower than that of experienced pilots on day 5 (t test; *p* = 0.0001, *p* = 0.005, respectively). On day 4, the peak oxyhemoglobin concentration was significantly lower than that of experienced pilots (*p* = 0.003) (Fig. 3, Fig. 6).

### Deoxyhemoglobin

#### Experienced pilots

In the experienced pilot group, the deoxyhemoglobin concentration in the left brain was -0.07 ± 0.08 µmol/L and in the right brain was -0.04 ± 0.05 µmol/L. The peak deoxyhemoglobin concentration was -0.20 ± 0.14 µmol/L in the left brain and -0.13 ± 0.06 µmol/L in the right brain. The duration of peak deoxyhemoglobin concentration was 200.71 ± 110.32 s in the left brain and 197.34 ± 84.15 s in the right brain.

#### Trainee pilots

In the trainee pilot group, the deoxyhemoglobin concentration in the left brain was -0.08 ± 0.06 µmol/L on day 1 and -0.02 ± 0.05 µmol/L on day 5 (*p* < 0.001). In the right brain it was -0.07 ± 0.11 µmol/L on day 1 and -0.03 ± 0.04 µmol/L on day 5 (*p* = 0.031). The peak deoxyhemoglobin concentration in the left brain was -0.19 ± 0.06 µmol/L on day 1 and -0.11 ± 0.09 µmol/L on day 5 (*p* = 0.002). The value on day 1 was significantly different than that of experienced pilots (t test, *p* = 0.019). The peak deoxyhemoglobin concentration in the right brain was -0.19 ± 0.11 µmol/L on day 1 and -0.12 ± 0.07 µmol/L on day 5 (*p* = 0.003). The value on day 1 was significantly different than that of experienced pilots (t test; *p* = 0.009). The duration of peak deoxyhemoglobin concentration in the left brain was 305.26 ± 118.09 s on day 1 and 203.14 ± 113.78 s on day 5 (*p* = 0.002). In the right brain it was 271.19 ± 133.28 s on day 1 and 202.39 ± 108.27 s on day 5 (*p* = 0.005). After repeated simulations, the mean and peak deoxyhemoglobin concentrations on day 5 were markedly lower than those of experienced pilots (t test; mean, *p* = 0.008; peak, *p* = 0.010). On day 4, the peak concentration in the right brain was also significantly lower than that of experienced pilots (*p* = 0.032) (Fig. 2, Fig. 5).

#### Experienced drivers

In the experienced driver group, the deoxyhemoglobin concentration in the left brain was -0.05 ± 0.07 µmol/L on day 1 and -0.01 ± 0.07 µmol/L on day 5 (*p* = 0.049). In the right brain it was -0.04 ± 0.07 µmol/L on day 1 and -0.00 ± 0.06 µmol/L on day 5 (*p* = 0.026). The peak deoxyhemoglobin concentration in the left brain was -0.16 ± 0.09 µmol/L on day 1 and -0.23 ± 0.14 µmol/L on day 4 (*p* = 0.017). In the right brain, it was -0.24 ± 0.11 µmol/L on day 2 and -0.16 ± 0.13 µmol/L on day 5 (*p* = 0.002). Notably, the mean and peak deoxyhemoglobin concentration in the left brain on the 5^th^ mission was significantly lower than that of experienced pilots (t test; *p* = 0.004, *p* = 0.040, respectively).

The duration of peak deoxyhemoglobin concentration in the left brain was 310.30 ± 173.11 s on day 1 and 143.54 ± 91.93 s on day 5 (*p* < 0.001). In the right brain it was 364.90 ± 231.79 s on day 1 and 140.12 ± 86.62 s on day 5 (*p* < 0.001). Notably, the peak duration in the left brain and the peak value and peak duration of deoxyhemoglobin concentration in the right brain during the first flight mission in the experienced driver group was significantly greater than that of experienced pilots (t test; *p* = 0.006, *p* = 0.015, *p* = 0.001, respectively).

After repeated simulations, on day 5 the mean concentration and the duration of peak concentration in the right brain in the experienced driver group were significantly lower than in the experienced pilot group (mean, *p* = 0.009; peak time duration, *p* = 0.014). On day 4, the peak concentration in the right brain was significantly lower than in the experienced pilot group (*p* = 0.045) (Fig. 3, Fig. 6).

### Correlation between mental workload and flight performance with flight task parameters

Mental workload was significantly correlated with all flight task parameters, except for preparation time (Table 1). Flight performance was significantly correlated with the flight task parameters of temporal demand (*r* = 0.308, *p* < 0.001), effort level (*r* = 0.218, *p* = 0.013), and task duration (*r* = 0.7728, *p* < 0.001) (Table 2).

**Table 1:** Pearson correlation analysis between mental workload and flight task parameters

	**Mental workload**		
Variable	Number	Pearson correlation coefficient	*p*
Flight performance	128	0.307	< 0.001
Physical workload	130	0.672	< 0.001
Temporal demand	130	0.704	< 0.001
Task effectiveness	130	0.643	< 0.001
Effort levels	130	0.694	< 0.001
Frustration	130	0.477	< 0.001
Preparation time	130	-0.090	0.309
Task duration	130	0.414	< 0.001
Total duration of preparation and task	130	0.366	< 0.001

**Table 2:** Pearson correlation between flight performance and flight task parameters

	**Flight performance**		
Variable	Number	Pearson correlation coefficient	*p*
Mental workload	128	0.307	< 0.001
Physical workload	128	0.159	0.073
Temporal demand	128	0.308	< 0.001
Task effectiveness	128	0.165	0.062
Effort level	128	0.218	0.013
Frustration	128	0.008	0.932
Preparation time	128	0.026	0.771
Task duration	128	0.801	< 0.001
Total duration of preparation and task	128	0.772	< 0.001

### Correlation between mental workload and oxyhemoglobin and deoxyhemoglobin concentrations

The mean and peak oxyhemoglobin concentrations were negatively correlated with the mean and peak deoxyhemoglobin concentrations in the right and left brain of the trainee pilots (Supplementary Table 1). The peak oxyhemoglobin was positively correlated with the mean oxyhemoglobin concentration, and the peak deoxygenated hemoglobin was negatively related to the mean deoxyhemoglobin concentration of trainee pilots. Representative dynamic changes in deoxyhemoglobin and oxyhemoglobin in 2 trainee pilots are shown in Fig. 5 and 6.

Flight performance was significantly correlated with mean oxyhemoglobin concentration (*r* = 0.233, *p* = 0.008) and peak oxyhemoglobin concentration (*r* = 0.245, *p* = 0.005) in the left brain. Flight performance was also significantly correlated with the duration of peak oxyhemoglobin concentration in both the left brain (*r* = 0.517, *p* < 0.001) and the right brain (*r* = 0.562, *p* < 0.001) (Table 3). Flight performance was also significantly correlated with peak deoxyhemoglobin concentration (*r* = -0.184, *p* = 0.038), duration of peak deoxyhemoglobin concentration (*r* = 0.179, *p* = 0.044), and duration of peak total deoxyhemoglobin concentration in the left brain (*r* = 0.421, *p* < 0.001), and was significantly correlated with the duration of peak deoxyhemoglobin concentration (*r* = 0.533, *p* < 0.001) and duration of peak total hemoglobin concentration in the right brain (*r* = 0.465, *p* < 0.001).

**Table 3:** Pearson correlation between mental workload and oxyhemoglobin and deoxyhemoglobin concentrations

	**Left brain**			**Right brain**		
Variables	Number	Pearson correlation coefficient	*p*	Number	Pearson correlation coefficient	*p*
Mean oxyhemoglobin	128	0.233	0.008		0.027	0.765
Peak oxyhemoglobin	128	0.245	0.005		0.143	0.108
Duration of peak oxyhemoglobin	128	0.517	< 0.001		0.562	< 0.001
Mean deoxyhemoglobin	128	-0.167	0.059		-0.076	0.396
Peak deoxyhemoglobin	128	-0.184	0.038		-0.104	0.242
Duration of peak deoxyhemoglobin	128	0.611	< 0.001		0.533	< 0.001
Mean total hemoglobin	128	0.179	0.044		-0.016	0.855
Peak total hemoglobin	127	0.175	0.048		0.113	0.203
Duration of peak total hemoglobin	128	0.421	< 0.001		0.465	< 0.001

## Discussion

In this study we examined flight performance and brain hemoglobin, oxyhemoglobin, and deoxyhemoglobin concentrations in pilot trainees, experienced pilots, and experienced drivers using a computer simulation flying experience. There were a number of important findings. 1) In trainee pilots and experienced drivers, after completing 5 simulations flight time was reduced, brain oxygen consumption was decreased, and learning was improved. 2) Changes in oxygen consumption in the left brain were more significant than those in the right brain, which is related to the function of the left frontal lobe as a sensory center. Spatial memory and working memory localize at the hippocampus and amygdala in the prefrontal lobe. 3) Most measured parameters were lower in the experienced driver group than in the experienced pilot group at the first simulation; however, at the 5^th^ simulation the values of most parameters in the experienced driver group were higher than in the experienced pilot group. These findings suggest that experienced drivers may have better potential to be trained as pilots than persons who have no experience in driving or piloting, and that this should be further studied.

There are currently no methods to measure mental workload in real-time, and thus determine the optimal level of mental workload and enhance the performance of pilots operating ROVs. Mental workload has been shown to affect objective and subjective aspects of pilot performance [6]. Despite the increasing use of ROVs and remotely piloted aircraft, including those used in military conflicts [17, 18], there is only scant knowledge of the level of mental workload required to operate these vehicles and mental workload correlates. In the current study we demonstrated that the mean oxyhemoglobin concentration reflects cerebral oxygen demand, the peak duration reflects the regulation of cerebral oxygen consumption. The mean and peak deoxyhemoglobin reflect brain oxygen consumption, and the peak duration reflects the regulation of oxygen consumption. With the repeated exercises (training), the brain's oxygen consumption decreases, the demand for oxygen decreases, and the body's demand for blood oxygen mobilization decreases. With the repeated exercises, the performance of the test subjects improved, and both the oxygen demand and oxygen consumption decreased. The experienced pilots served as positive controls, and initially cerebral oxygen demand and oxygen consumption were significantly higher for trainee pilots and experienced drivers. However, after the repeated trainings their oxygen demand and oxygen consumption decreased.

It has been reported that experienced drivers have better spatial positioning ability than non-drivers; however, drivers and non-drivers have similar stereo-positioning ability [19]. Through training, the trainee pilots achieved similar levels of flight performance to those of experienced drivers. We found that the oxyhemoglobin and deoxyhemoglobin concentrations on day 1 or 2 was markedly higher than that on day 4 or 5, suggesting a decline in mental workload. The peak oxyhemoglobin and deoxyhemoglobin concentration and peak duration also decreased, especially the oxyhemoglobin concentration, indicating that upon an increase in mental workload, brain tissues mobilize a systemic oxygen supply. Notably, the left brain showed greater changes in blood oxygen than the right brain, which may be due to right hand dominance in the study cohort, leading to greater oxygen demand in the left hemisphere. With training, the pilot trainees and experienced drivers were able to operate the aircraft well in subsequent days, at which point there were declines in the various parameters examined. These parameters were noticeably lower compared to those on the 4^th^ or 5^th^ flight missions. Most trainee pilots and experienced drivers were adept at flying remotely on the 4^th^ mission. They might have become overconfident and eager to complete the assigned task on the 5^th^ mission and took off less than 5 s for a destination and thus had to restart the mission. This may explain the decline in performance scores on the 5^th^ mission.

Oxyhemoglobin and deoxyhemoglobin concentrations change with changes in brain activity [20]. fNIRS is a non-invasive method for measuring prefrontal cortex activity, and suitable for investigating changes in cerebral oxygenation during performance of tasks [21, 22]. Durantin et al. used fNIRS to study 12 volunteers who followed a dynamic target with their aircraft using a computer-based system [8], and found that fNIRS was sensitive to different levels of mental workload. Kobayashi and Miyamoto demonstrated continuous changes in cerebral oxygen levels in fighter pilots during flight using fNIRS [23]. León-Carrión and León-Domínguez showed that levels of oxyhemoglobin rose and those of deoxyhemoglobin declined in response to increased mental activity [24]. In the current study, we tracked specific changes in oxyhemoglobin and deoxyhemoglobin in the brains of trainee pilots remotely operating aircraft, and found that oxyhemoglobin levels became lower with decreasing mental workload while deoxyhemoglobin levels increased with decreasing mental workload. However, our correlation analysis revealed no correlation between mean and peak oxyhemoglobin or deoxyhemoglobin and mental workload. On the other hand, the mental load of the trainee pilots positively correlated with the duration of peak oxyhemoglobin concentration, and negatively correlated with the duration of peak deoxyhemoglobin concentration, suggesting that the duration of peak oxyhemoglobin or deoxyhemoglobin could be an indicator of mental workload in trainee pilots remotely operating aircraft, and provide a method to monitor the mental workload of trainee pilots.

Mental workload can have a direct impact on the performance of pilots. An optimal level of mental workload facilitates flight performance, while mental overload may negatively affect flight performance and cause psychological symptoms, such as frustration. We found that the mental workload of the trainee pilots was significantly correlated with frustration level, and as the mental workload of the trainee pilots decreased, their frustration levels also decreased.

There are several limitations to this study that should be considered. Previous studies have shown that varying task loads or task difficulties can affect the flight performance of pilots. In the current study, we did not vary the difficulty of flight tasks. However, we did investigate changes over 5 days of flight performance, which reinforces spatial memory and memory retrieval and flight maneuvering through an iterative process. Temporal domain analysis is the most commonly used method in the field of fNIRS, and thus was used in this study. However, fNIRS data in the frequency domain contains a great deal of information but was not the subject of the current study. In future studies, we plan to analyze fNIRS data in frequency domain in order to further examine hemodynamic responses. On the other hand, a strength of this study ***was*** the use of fNIRS, which showed that with increased learning, the mental workload of remotely operating the aircraft decreased, and changes were observed in flight time, mental workload, and oxyhemoglobin and deoxyhemoglobin over time. Lastly, we only monitored subjects with fNIRS, and did not monitor physiological responses such as heart rate variability, blood pressure, eye movements, and changes in pupil size, which are known to correlate with mental workload and task performance. However, these parameters have not been shown to be of specific value in evaluating mental workload in previous studies, which is why they were not included in the current study. In addition, wearing a monitor to examine these parameters could interfere with flight performance.

Although there are certain limitations, the current study provides valuable insights in investigation of mental workload of trainee pilots of remotely operated aircraft. To make the mental workload evaluation of pilot more applicable and reliable, advanced techniques and processing methods will be utilized in further study.

## Conclusions

In conclusion, to the best of our knowledge this was the first study to assess the mental workload of trainee pilots remotely operating aircraft using fNIRS in a simulated flight experience. We demonstrated that the duration of peak oxyhemoglobin and deoxyhemoglobin correlated with the mental workload of remotely operating the aircraft. Our results suggest that hemodynamic parameters such as duration of peak oxyhemoglobin and deoxyhemoglobin concentration are promising surrogate markers for mental workload of trainee pilots remotely operating aircraft. Monitoring these parameters may help to establish a simplified scheme for optimizing flight performance based on the mental workload of a trainee pilot.

## Supplementary Information

**Additional file 1:** **Supplementary Table 1.** Correlation among hemodynamic parameters of thetrainee pilots.

## Data Availability

The datasets used and/or analyzed during the current study are available from the corresponding author on reasonable request.
