# Supplementary material for: Assessment of the mental workload of trainee pilots of remotely operated aircraft using functional near-infrared spectroscopy
Source: BMC Neurol. 2022 Apr 30;22:160. doi: 10.1186/s12883-022-02683-5 (PMC9055770; doi:10.1186/s12883-022-02683-5)
Supplement: Supplementary file 1 — Additional file 1: Supplementary Table 1. Correlation among hemodynamic parameters of thetrainee pilots. [file 12883_2022_2683_MOESM1_ESM.doc]

**Supplementary Table 1** Correlation among hemodynamic parameters of the trainee pilots

|  |  |  |  | **Left brain** |  |  |
| --- | --- | --- | --- | --- | --- | --- |
|  |  | **Mean oxyhemoglobin** | **Peak oxyhemoglobin** | **Peak oxyhemoglobin duration** | **Mean deoxyhemoglobin** | **Peak deoxyhemoglobin** |
|  | **Mean oxyhemoglobin** |  |  |  |  |  |
|  | N | 130 | 130 | 130 | 130 | 130 |
|  | Pearson | 1 | .791^**^ | .303^**^ | -.635^**^ | -.619^**^ |
|  | P |  | <.001 | <.001 | <.001 | <.001 |
|  | **Peak oxyhemoglobin** |  |  |  |  |  |
|  | N | 130 | 130 | 130 | 130 | 130 |
|  | Pearson | .791^**^ | 1 | .385^**^ | -.381^**^ | -.599^**^ |
|  | P | <.001 |  | <.001 | <.001 | <.001 |
|  | **Peak oxyhemoglobin duration** |  |  |  |  |  |
|  | N | 130 | 130 | 130 | 130 | 130 |
|  | Pearson | .303^**^ | .385^**^ | 1 | -.301^**^ | -.409^**^ |
|  | P | <.001 | <.001 |  | .001 | <.001 |
|  | **Mean deoxyhemoglobin** |  |  |  |  |  |
|  | N | 130 | 130 | 130 | 130 | 130 |
|  | Pearson | -.635^**^ | -.381^**^ | -.301^**^ | 1 | .843^**^ |
|  | P | <.001 | <.001 | .001 |  | <.001 |
|  | **Peak deoxyhemoglobin** |  |  |  |  |  |
| **Left brain** | N | 130 | 130 | 130 | 130 | 130 |
|  | Pearson | -.619^**^ | -.599^**^ | -.409^**^ | .843^**^ | 1 |
|  | P | <.001 | <.001 | <.001 | <.001 |  |
|  | **Peak deoxyhemoglobin duration** |  |  |  |  |  |
|  | N | 130 | 130 | 130 | 130 | 130 |
|  | Pearson | .209^*^ | .270^**^ | .867^**^ | -.320^**^ | -.347^**^ |
|  | P | .017 | .002 | <.001 | <.001 | <.001 |
|  | **Mean total hemoglobin** |  |  |  |  |  |
|  | N | 130 | 130 | 130 | 130 | 130 |
|  | Pearson | .834^**^ | .749^**^ | .174^*^ | -.105 | -.199^*^ |
|  | P | <.001 | <.001 | .049 | .239 | .024 |
|  | **Peak total hemoglobin** |  |  |  |  |  |
|  | N | 129 | 129 | 129 | 129 | 129 |
|  | Pearson | .587^**^ | .900^**^ | .264^**^ | -.054 | -.247^**^ |
|  | P | <.001 | <.001 | .002 | .542 | .005 |
|  | **Peak total hemoglobin duration** |  |  |  |  |  |
|  | N | 130 | 130 | 130 | 130 | 130 |
|  | Pearson | .377^**^ | .494^**^ | .824^**^ | -.219^*^ | -.381^**^ |
|  | P | <.001 | <.001 | <.001 | .012 | <.001 |
|  | **Mean oxyhemoglobin** |  |  |  |  |  |
|  | N | 130 | 130 | 130 | 130 | 130 |
|  | Pearson | .615^**^ | .416^**^ | .216^*^ | -.476^**^ | -.468^**^ |
|  | P | <.001 | <.001 | .013 | <.001 | <.001 |
|  | **Peak oxyhemoglobin** |  |  |  |  |  |
|  | N | 130 | 130 | 130 | 130 | 130 |
|  | Pearson | .419^**^ | .341^**^ | .264^**^ | -.256^**^ | -.331^**^ |
|  | P | <.001 | <.001 | .002 | .003 | <.001 |
|  | **Peak oxyhemoglobin duration** |  |  |  |  |  |
|  | N | 130 | 130 | 130 | 130 | 130 |
|  | Pearson | .234^**^ | .276^**^ | .783^**^ | -.252^**^ | -.362^**^ |
|  | P | .007 | .001 | <.001 | .004 | <.001 |
|  | **Mean deoxyhemoglobin** |  |  |  |  |  |
|  | N | 130 | 130 | 130 | 130 | 130 |
|  | Pearson | -.428^**^ | -.214^*^ | -.202^*^ | .515^**^ | .413^**^ |
| **Right brain** | P | <.001 | .015 | .021 | <.001 | <.001 |
|  | **Peak deoxyhemoglobin** |  |  |  |  |  |
|  | N | 130 | 130 | 130 | 130 | 130 |
|  | Pearson | -.467^**^ | -.377^**^ | -.314^**^ | .471^**^ | .523^**^ |
|  | P | <.001 | <.001 | <.001 | <.001 | <.001 |
|  | Peak **deoxyhemoglobin** duration |  |  |  |  |  |
|  | N | 130 | 130 | 130 | 130 | 130 |
|  | Pearson | .215^*^ | .215^*^ | .786^**^ | -.262^**^ | -.294^**^ |
|  | P | .014 | .014 | <.001 | .003 | .001 |
|  | **Mean total hemoglobin** |  |  |  |  |  |
|  | N | 130 | 130 | 130 | 130 | 130 |
|  | Pearson | .436^**^ | .340^**^ | .122 | -.225^**^ | -.279^**^ |
|  | P | <.001 | <.001 | .167 | .010 | .001 |
|  | **Peak total hemoglobin** |  |  |  |  |  |
|  | N | 130 | 130 | 130 | 130 | 130 |
|  | Pearson | .260^**^ | .214^*^ | .188^*^ | -.086 | -.138 |
|  | P | .003 | .014 | .032 | .331 | .116 |
|  | **Peak total hemoglobin duration** |  |  |  |  |  |
|  | N | 130 | 130 | 130 | 130 | 130 |
|  | Pearson | .281^**^ | .319^**^ | .736^**^ | -.225^**^ | -.336^**^ |
|  | P | .001 | <.001 | <.001 | .010 | <.001 |
